# An epidemiological and clinical analysis of craniomaxillofacial fibrous dysplasia in a Chinese population

**DOI:** 10.1186/1750-1172-7-80

**Published:** 2012-10-17

**Authors:** Jie Cheng, Yanling Wang, Hongbo Yu, Dongmiao Wang, Jinhai Ye, Hongbin Jiang, Yunong Wu, Guofang Shen

**Affiliations:** 1 Department of Oral and Maxillofacial Surgery, Affiliated Stomatological Hospital, Nanjing Medical University, Jiangsu, China; 2 Department of Oral and Maxillofacial Surgery, Shanghai Ninth People’s Hospital, Shanghai Jiao Tong University School of Medicine, Shanghai, China; 3 , Hanzhong Road 136, Nanjing, 210029, PR China

**Keywords:** Craniomaxillofacial fibrous dysplasia, Epidemiology, Diagnosis, Treatment

## Abstract

**Background:**

Craniomaxillofacial fibrous dysplasia (FD) is a benign bone lesion characterized by facial disfigurement and functional impairment. The aim of this study was to characterize the epidemiological and clinical features of craniomaxillofacial FD by presenting data from a representative Chinese population during a 15-year period (1994–2009).

**Method:**

The craniomaxillofacial disease registries of two Chinese tertiary referral hospitals (Shanghai Ninth People’s Hospital and Stomatological hospital of Jiangsu Province) were searched and reviewed to collect relevant information for patients with craniomaxillofacial FD between Jan.1994 and Dec.2009. All included cases were further analyzed with regard to associated epidemiological and clinicopathological variables.

**Results:**

A total number of 266 cases with definitive diagnosis were identified with 219 primary cases and 47 recurrent cases. There were 111 males and 155 females with a male to female ratio of 0.716:1. They were clinically categorized into three groups: monostotic (71.1%), polysotic (27.4%) and Albright syndrome (1.5%). Maxilla alone or with adjacent bones was the most common affected site. The serum alkaline phosphatase (ALP) in patients was much higher than that in healthy control, whereas comparable between primary patients and recurrent ones. Three patients (3/266, 1.1%) with polysotic lesions underwent spontaneous malignant transformation into osteosarcoma. The majority of patients underwent conservative surgery, while the others received radical resection with or without reconstruction.

**Conclusions:**

Craniomaxillofacial FD is a rare bony disorder with defined epidemiological and clinicopathological features in Chinese population. Further investigations are warranted to establish the optimized timing, treatment strategy and prognostic prediction for this clinical entity.

## Introduction

Fibrous dysplasia (FD) is a benign bone lesion characterized by the replacement of normal bone structure with abnormal fibro-osseous connective tissue [1]. It accounts for about 2-3% of bone-derived tumors and usually occurs throughout the skeletons around the body with a predilection for craniomaxillofacial bones [2]. Craniomaxillofacial FD usually begins in childhood and slowly progresses through puberty, resulting in devastating facial deformities and functional deficits [3]. These lesions develop not only in single bone (monstotic) but also in multiple bones (polystotic), sometimes concomitantly associated with skin pigmentation and endocrine disturbances (McCune-Albright syndrome). Activating mutations of the alpha subunit of stimulatory G protein gene(GNAS) at Arg^201^ and increased proliferation and inappropriate differentiation in the osteoblastic cells has been identified to be implicated in the FD pathogenesis [4].

In craniomaxillofacial region, maxilla is more commonly affected than mandible and usually involves adjacent bones such as sphenoid, zygoma and frontal bone. These patients often present with a painless, slowly enlarging and hard swelling in affected bones. Its overgrowth tends to result in facial asymmetry, disfigurement, malocclusion, orbital dystopia and exophthalmos, largely depending on anatomical sites and structure involved [5]. Radiographic characteristics include cystic, sclerotic or pagetoid, often a typical ground-glass appearance with ill-defined margins. Under the microscope, these lesions usually show irregularly shaped trabeculae of immature woven bone in compact stroma of interlacing collagen fibers without sharp demarcation. Various treatment strategies are available for these lesions, ranging from watchful expectancy for arrested growth after puberty to conservative therapy (recontouring, curettage or trimming), to radical resection followed by immediate reconstruction. The prognosis for patients with craniomaxillofacial FD is usually favorable, although spontaneous malignant transformation is rarely reported in the literature [6].

However, the lack of information regarding the epidemiological and clinical characteristics of craniomaxillofacial FD complicates in-depth understanding of these entities and impedes advances for their diagnosis and treatment. Furthermore, to the best of our knowledge, no information about the epidemiological data regarding craniomaxillofacial FD from Chinese population has been reported thus far, which necessitates an epidemiological and clinical investigation into this disease. In the present study, we retrospectively reviewed and presented the epidemiological and clinical data of craniomaxillofacial FD from two Chinese tertiary referral hospitals over the past 15 years (1994–2009) in order to highlight the demographic, clinical and pathological characteristics in a large representative Chinese population. These data might also provide valuable information for comparison with other epidemiological studies from different geographical site and races.

## Patients and methods

Both of craniomaxillofacial disease registries of Shanghai Ninth People’s Hospital and Stomatological Hospital of Jiangsu Province were reviewed to retrieve relevant information about patients with craniomaxillofacial FD receiving surgeries during the past 15 years (1994–2009). Inclusion criteria comprised the patients with detailed data including precise location, definitive diagnosis by clinical manifestations, radiographic findings and histopathology. The medical charts or records of these enrolled patients were retrieved and subjected to further review to obtain information regarding patient demographics, clinicopathological features, treatment modalities and outcomes. This clinical retrospective study was approved by both institutional academic committees and performed in accordance with guidelines of the Declaration of Helsinki. Informed consent was obtained from patients or their guardians.

The definitive diagnosis of craniomaxillofacial FD was made largely based on clinical symptoms and signs, radiographic features and postoperative pathological findings. In this study, diseases affecting two or more segments of one bone in the craniofacial region were classified as monostotic lesions, while abnormal growths involving multiple bones in this area were defined as polyostotic lesions. The patients with characteristic triad conditions were diagnosed as McCune-Albright syndrome. Additionally, the recurrent cases were defined as the patients sought to receive one more treatment following their initial surgeries.

All patients included here were treated by surgery which can be divided into two main categories: conservative surgery (partial resection: trimming, recontouring and curettage) and radical surgery (complete resection and/or simultaneous reconstruction). The aims of surgical treatment for these patients were to correct the functional problems (such as optic nerve compression with progressive deterioration of vision when orbit involved and severe skeletal malocclusion when jaws affected) and achieve aesthetic improvement when facial deformities became evident. Therefore, the main surgical indications for most patients in our cohort were severe facial deformities, devastating functional disturbance and avoidance of potential malignant transformation. These patients were recommended to participate in the long-term follow-up by clinical and radiographic examinations (CT scan) at regular intervals.

All relevant data available from every patient were collated and tabulated using Microsoft Excel. Descriptive statistical analysis was performed for the variables of interest. Difference between experimental groups was assessed by Student’s *t*-test using SPSS software package 13.0. *p*<0.05 was considered statistically significant.

## Results

Our registry searches have identified a total number of 266 patients with craniomaxillofacial FD treated at both institutions between 1994 and 2009 according to the preset inclusion criteria. There were 219 primary cases and 47 recurrent cases, each accounting for 82.3% (219/266) and 17.7% (47/266), respectively. Most recurrent cases were treated previously in other clinics and then referred to our institutions for further treatment mainly due to apparent disease regrowth and/or unsatisfied facial profile. All these patients were treated by conservative or radical surgeries and definitive diagnoses were further confirmed by postoperative histopathological evidence.

**Figure 1:**
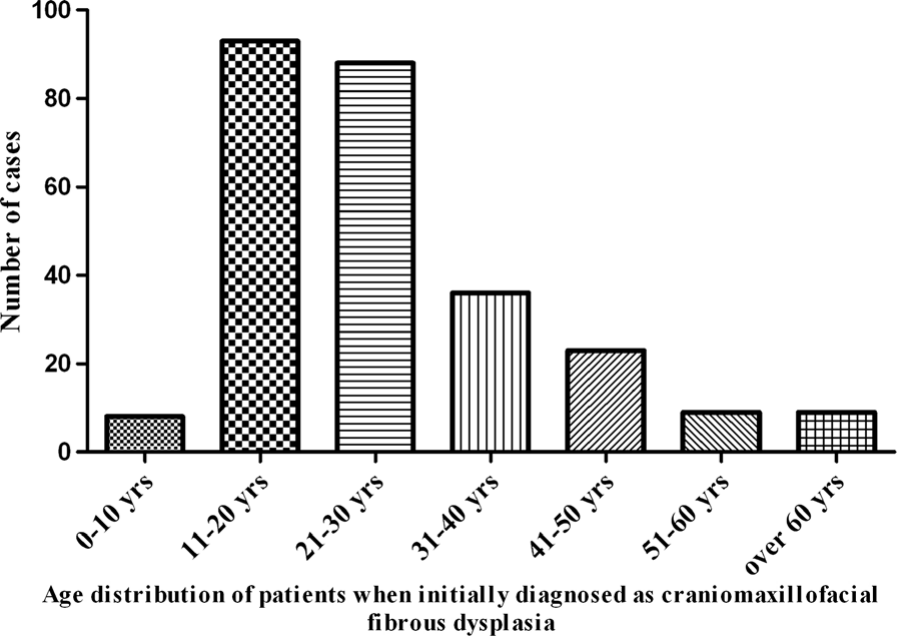
Age distribution of patients when initially diagnosed as craniomaxillofacial FD.

**Figure 2:**
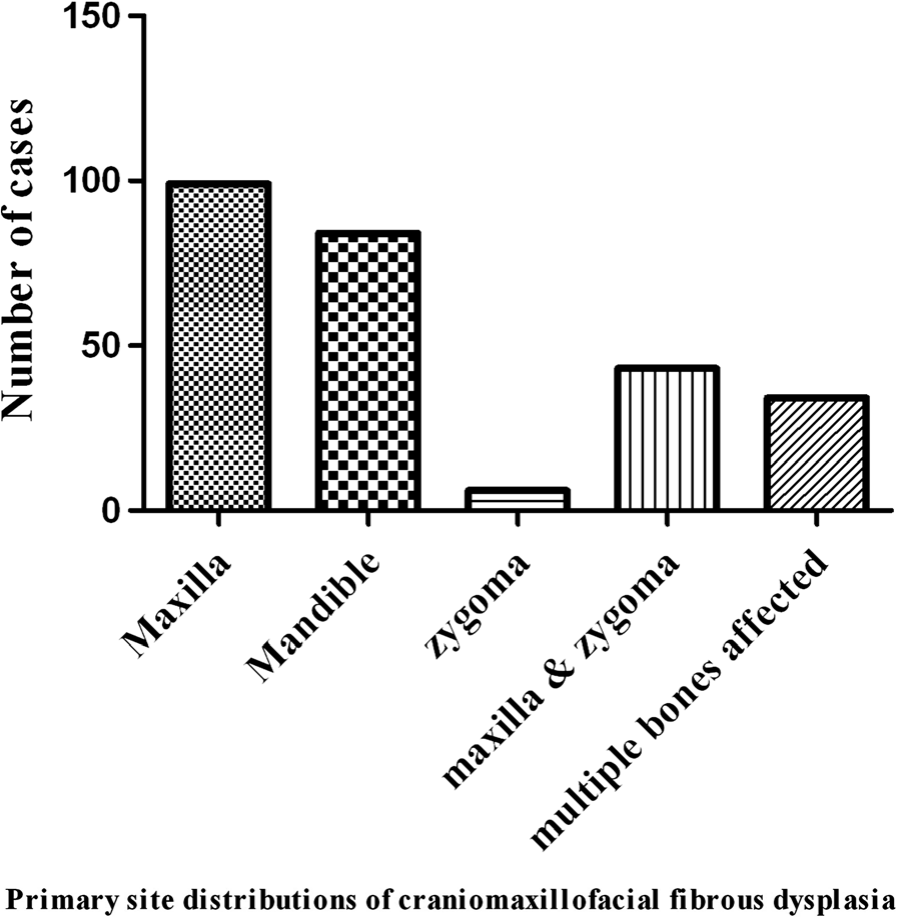
Primary site distribution of patients with craniomaxillofacial FD.

**Figure 3:**
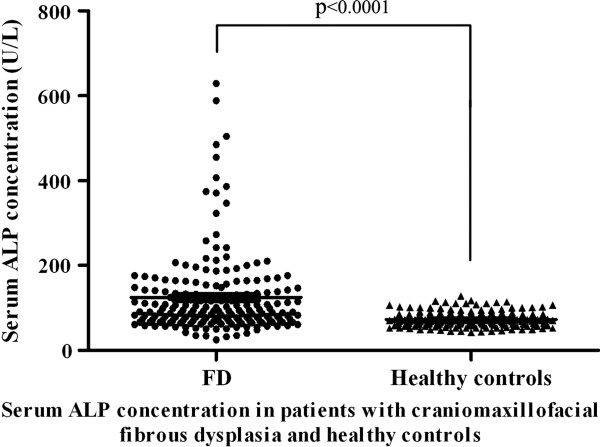
Serum ALP concentration in patients with craniomaxillofacial FD and healthy controls.

Among 266 patients, there were 111 males and 155 females with a male to female ratio of 0.716:1, an indicative of a female predominance. The average age of these patients at the time of receiving surgeries was 27.1 years (range 9–70 years). Age distribution as shown in Figure 1 indicated that most patients underwent surgical treatment at the second and third decades of life, each accounting for 34.9% (93/266) and 33.1% (88/266). Moreover, the time intervals between initial diagnosis and surgical treatment ranged from a few weeks to more than 20 years. Approximately 50% of these patients underwent surgery less than 5 years after initial diagnosis. With regard to the primary sites of disease, 109 patients involved the left side, 138 affecting the right side and 19 affecting both sides. According to the X-ray and CT scan images prior to surgery, maxilla was identified as the most common bone involved, followed by mandible, zygoma, zygomaticomaxillary complex and multiple bones in descending sequence as shown in Figure 2. Clinical subtypes of these patients were then accordingly categorized as monostotic lesions (189, 71.1%), polysotic lesions (73, 27.4%) and Albright syndrome (4, 1.5%). The concentrations of serum ALP (alkaline phosphatase) available for 192 patients with craniomaxillofacial FD ranged from 34 to 629 U/L with an average 125.1 U/L, which were significantly higher than those in both age-and sex-matched healthy controls as indicated in Figure 3 (average 73.1 U/L).

**Figure 4:**
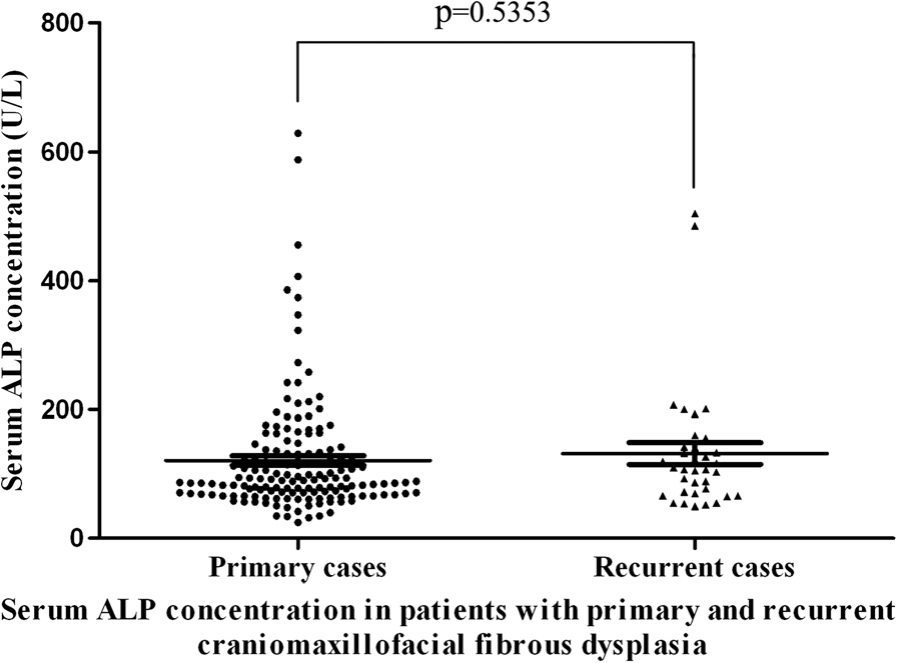
Serum ALP concentration in patients with primary and recurrent FD.

As regard to these recurrent cases, they all have received conservative surgeries before they sought one more treatment at our institutions, whereas no recurrence was found in patients who previously underwent radical resection. Fourteen were males and thirty-three were females. The mean age of these recurrent patients was 28.6 years (16–69 years). They can be categorized as 28 monostotic lesions, 18 polystotic lesions and 1 Albright syndrome. The time intervals between initial surgery and further surgical treatment at our tertiary referral hospitals ranged from 6 to 192 months (average 63.7 months). The average serum ALP was 131.7 U/L in such patient group, which was much higher than healthy controls(average 73.1 U/L, *P*<0.01). Moreover, such parameters in recurrent cases appeared a little higher than those in primary cases, although no statistical significant difference was found (Figure 4).

Among 266 patients, 3 patients (3/266, 1.1%) were found to undergo spontaneous malignant transformation of fibrous dysplasia into osteosarcoma which were confirmed by postoperative pathological examinations as evidenced by the existence of both fibrous dysplasia and osteosarcoma. All malignant diseases occurred in maxilla and adjacent zygoma and sphenoid. The clinical subtypes of these patients were initially categorized as polyostotic. Medical records indicated that no patients had history of prior radiotherapy and the concentrations of serum ALP were 65, 323, 763, respectively. Two patients died of tumor and one remained alive after radical resection during the follow-up (6-23months).

The clinical presentation of craniomaxillofacial fibrous dysplasia primarily depends the site, extent and nature of these bone lesions. The most common clinical manifestation was gradual and painless swelling and then resulting facial deformities and disfigurements and malocclusion. Some patients can also present with proptosis, diplopia, hearing loss, nasal obstruction and loss of vision when various structures and areas were affected. The plain radiographic features of craniomaxillofacial fibrous dysplasia were non-specific and often presented as radiolucent lytic lesion with homogenous ground-glass appearance and ill-delineated borders. Computed tomography (CT) provides better assessment of the extent of bone involved. Both plain radiography and CT scan were essential for diagnosis, treatment planning and postoperative monitoring for this clinical entity in our diagnosis-treatment regime.

All patients with craniomaxillofacial FD were received surgeries either conservative or radical one largely depending on the extent of bone involvement and patients’ choices as listed in Table 1. The majority of patients (87.9%) underwent conservative surgery including trimming and recontouring of the affected bones, while the others received radical resection with or without reconstruction. No radiotherapy and chemotherapy was routinely given prior or after operation. These patients were routinely received regular follow-up to examine the disease regrowth and potential spontaneous malignant transformation.

**Table 1:** Treatment procedures for patients with craniomaxillofacial FD

**Treatment**	**Patients No.**
**Conservative surgery**	
Trimming & recontouring	214
Trimming + orthoganthic surgery	4
Navigation-assisted bone trimming	12
**Radical surgery**	
Total resection without reconstruction	16
Total resection with Ti-plate/mesh reconstruction	4
Total resection with free bone graft	4
Total resection with flap reconstruction^*^	12

Note: * flap reconstructions included 10 vascularized fibular flap, 1 vascularized iliac flap and 1 costochondral graft.

## Discussion

Fibrous dysplasia is a benign and rare bone disorder that often affects the craniofacial skeleton [7]. It usually cause significant morphological and functional disturbance, such as malocclusion, devastating facial aesthetics, et al. The main treatment goal and challenge for these patients with craniomaxillofacial FD is to restore normal facial cosmetics and correct the associated deformities [8]. Therefore, the comprehensive understanding of this clinical entity might be the prerequisite for its early detection, accurate diagnosis, proper management and prognostic prediction. To the best of our knowledge, this study might for the first time provide comprehensive epidemiological and clinicopathological information about craniomaxillofacial FD in patients of Chinese origin, which is of great value for clinicians to manage this clinical entity efficiently and properly.

Previous reports have revealed that gender distribution of fibrous dysplasia was inconsistent with varied ratios among different populations. Kruse A. et al. reported a female to male ratio 3:1 in their Swiss series of 8 craniomaxillofacial FD [3]. However, several others reported that it appeared to occur equally in males and females, indicating no sex predilection [9]. This discrepancy may be resulted from limited number of patients examined or disease susceptibility among different races. However, in this large number of Chinese patients, female preponderance was identified evidently. Fibrous dysplasia often begins in childhood and progresses through puberty and arrests in adolescence [10]. Our data indicated that the majority of patients sought treatment in their second and third decade of life**.** However, growth arrest for fibrous dysplasia seemed unpredictable and continued growth during the adulthood was frequently observed in the clinic, which also can be evidenced from the fact that 43 patients (18.2%) with over 40-years-old sought surgical treatments.

The etiology and molecular pathogenesis of abnormal fibrous-osseous process in fibrous dysplasia has been gradually unraveled. Activating mutation of the Gs-a protein and increased interleukin-6 synthesis in osteoprogenitor cells has been implicated in the initiation and progression of these lesions [11,12]. Although this entity was commonly regarded as benign, malignant transformation to osteosarcoma, fibrosarcoma or chondrosarcoma was sporadically reported in decreased order of frequency, approximately accounting for 0.4-1% percentage of all cases [13]. Prior radiotherapy was suggested to be associated with malignant degeneration [14]. In this patient group, three patients with polysotic lesions (maxilla, zygoma and sphenoid affected) were identified with sarcomatic transformation in the pre-existing fibrous lesions. However, no prior radiological exposure to these patients was recorded. Thus, the roles of radiotherapy in malignant transformation remain further investigation.

Although fibrous dysplasia may involve any bone throughout the body, several bones including maxilla, mandible, sphenoid, frontal and temporal bone were usually affected alone or together. In the present study, the relative numbers of patients with monostotic and polyostotic disease are generally similar with literature report, which the former accounted for 70% and the later accounted for 30% [9]. Maxilla was identified as the most commonly involved bone in the monstotic lesions. These polystotic lesions usually affected the adjacent bones separated by sutures especially the zygoma and sphenoid.

The clinical presentation of craniofacial fibrous dysplasia varies largely depending on the site, duration and extent of lesion. Generally, it usually presents as a slowly-enlarging, painless, immobile and hard swelling, thereby resulting in facial asymmetry and disfigurement, malocclusion Visual impairment and hearing loss occur when the vital neurovascular structures are compressed by enlarged lesions. The disease onset is often slow and gradual, so that varied intervals between onset and treatment were observed. A fraction of patients only sought treatments when obvious facial deformities or functional disturbance were present. Suddenly accelerated growth and onset of pain in a longstanding quiescent lesion may indicate the possibility of malignant transformation and demands prompt examination and vigorous therapy when necessary.

Diagnosis of fibrous dysplasia is based on clinical examination, radiographic and histopathological findings. Therapeutic options for craniomaxillofacial fibrous dysplasia include wait and see, conservative surgery and radical surgery [8]. Nevertheless, no consensus about optimal treatment strategy has been well established and remains a matter of controversy. Surgical indications usually include correction of craniofacial deformities and treatment of functional disturbance. The timing for surgical intervention for craniomaxillofacial FD has always been debated, especially in growing children [15]. In earlier years, surgeons were recommended to wait and defer all surgical procedures until the disease might become stabilized or arrested after puberty. However, the unpredictable growing process of lesions and gradually devastating facial form and function imposed to young children made such procrastination unacceptable. Early surgical intervention becomes necessary and logical at such instances that these diseases obviously affect patients’ facial esthetic and functions. Some authors advocated conservative shaving and curettage as the principal treatment of choice, largely due to slowly growing process, possible growth arrest in adolescence and associated morbidities with aggressive surgery [16,17]. Recently, several authors argued that conservative approach can’t completely eliminate the diseased bone and a high rate of recurrence or regrowth was reported especially in younger patients. They emphasized that radical resection and reconstruction for craniomaxillofacial FD was feasible and logical, which permitted the complete removal of lesions and avoided risk of malignant changes [18,19]. However, in our patient series, the overwhelming majority underwent conservative surgery including trimming, curettage and recontouring, whereas 36 patients (13.5%) received radical resection with or without simultaneous reconstruction. Although the consensus about treatment indications for craniomaxillofacial FD has not been reached, our indications for surgery usually included severe facial disfigurement (evident facial swelling and asymmetry) and functional disturbance (continuous visual loss and severe malocclusion) to achieve esthetic facial profile and functional improvement. We believed that the choice between conservative and radical surgery depends on the following factors: affected site, growth rate, cosmetic and functional impairment, patient preference and surgeon’s experience and multi-disciplinary team approach. The treatment option for each patient should be individualized to weigh the pro and cons of both surgical approaches. We preferred the conservative surgery mainly owing to the benign nature, growth trajectory, and exceeding rarity of malignant transformation of fibrous dysplasia. More importantly, the latest surgical progresses especially the image-guided navigation-assisted surgery greatly improved the postoperative results of conservative surgery for patients as we and others reported before [20,21]. However, it’s clear that the conservative approach might not be suitable for all patients. When patients with multiple recurrences, severe facial disfigurement and local refractory osteomyelitis were encountered, radical surgery was indicated as treatment of choice. Following complete resection, several reconstruction options were applied including vascularized fibular and iliac flap to restore the facial profile and provide enough bone for staged osseointegrated dental implants.

As regard to prognostic factor for craniomaxillofacial FD, in addition to age at the time of surgery, the possibility of complete resection has been commonly emphasized. Recently, serum alkaline phosphatase (ALP) has been proposed to be associated with recurrence following conservative surgery and potential malignant transformation. It appeared as a reliable marker for predicting the progress and treatment outcome of craniomaxillofacial FD [22]. In our study, similar with previous reports, the level of ALP was much higher in patient than healthy controls. Additionally, levels of ALP in two patients with malignant transformation were 4 and 10 folds higher than healthy controls respectively. However, its levels in primary cases were comparable with that in recurrent cases. Collectively, our results support this notion that serum ALP is a useful biomarker for FD diagnosis, but its values in prognostic prediction still remain further validations.

In conclusion, craniomaxillofacial FD is a rare bone disease with defined epidemiological and clinical traits in Chinese population. The treatment of choice should be tailed to every patient. Further investigations are required to establish the optimized timing, treatment choice and prognostic predictors for patients with craniomaxillofacial FD.

## Competing interest

The authors declare that they have no competing interests.

## Authors' contributions

JC, YW and GS conceived this study and draft this manuscript. JC, YW, HY and DW performed the data collection and analysis. JY and HJ carried out statistical analyses. All authors read and approved the final manuscript.
